# Editorial

**Published:** 1891-01

**Authors:** 


KOCH'S LYMPH.—Nothing has as yet been definitely settled as to the real curative value of parataloid. It is now being experimented with by a number of leading American physicians. Few absolute cures are claimed. Cases of lupus have relapsed after apparent cures. Pasteur and other leading French scientists criticize the method severely. Just what the outcome of the discovery will be it is impossible to predict.

---

ATLANTA SOCIETY OF MEDICINE.—The following officers have been elected for the ensuing year: President, Floyd W. McRae, M. D.; Vice President, Arthur G. Hobbs, M. D.; Recording Secretary, J. A. Childs, M. D.; Treasurer, E. van Goidtsnoven, M. D.; Corresponding Secretary and Librarian, N. O. Harris, M. D.

---

THE CLINIC.—The *Clinic* is the title of a new monthly journal, edited by Messrs. Frank Park, R. E. Smith, H. S. Bruce, B. F. Hart and A. A. Barge, members of the graduating class of the Atlanta Medical College. It is a class paper and gives epitomes of the most important lectures and clinics, and besides contains many breezy items. THE JOURNAL extends to the clinic a hearty welcome, and hopes for it the success which its merit deserves.

---

THE MATTISON PRIZE. OPIUM ADDICTION AS RELATED TO RENAL DISEASE. A PRIZE OF FOUR HUNDRED DOLLARS.—With the object of advancing scientific study and settling a now mooted question, Dr. J. B. Mattison, of Brooklyn, offers a prize of $400 for the best paper on "Opium Addiction as Related to Renal Disease," based upon these queries:

Will the habitual use of opium, in any form, produce organic renal disease?

If so, what lesion is most likely?

What is the rationale?

The contest is to be open for two years from Dec. 1, 1890, to either sex, and any school or language.

The prize paper is to belong to the American Association for the Cure of Inebriety, and be published in a New York medical journal, *Brooklyn Medical Journal* and *Journal of Inebriety.*

Other papers presented are to be published in some leading medical journal, as their authors may select.

All papers are to be in possession of the Chairman of Award Committee, on or before January 1, 1893.

The Committee of Award will consist of Dr. Alfred L. Loomis, Pres. N. Y. Acad. of Medicine, Chairman; Drs. H. F. Formad, Phila.; Ezra H. Wilson, Brooklyn; Geo. F. Shrady, and Jos. H. Raymond, editor *Brooklyn Medical Journal.*

---

HYDRONAPHTHOL AS AN ANTISEPTIC.—Mr. Thos. H. Bryce (*British Med. Journal*, Nov. 22, 1890) reports the results of a series of carefully conducted experiments and investigations of the antiseptic value of hydronaphthol. He found it to be a very efficient antiseptic, but not available for general use on account of its insolubility in water.

---

FRACTURE OF LOWER END OF RADIUS.—Dr. John B. Roberts, (*Medical News*, Dec. 13, 1890) argues that splints are of very little value in fractures of the lower end of the radius and that as usually applied they are positively harmful. He considers reduction the essential part of the treatment, and deems splints in manageable patients useless. Where a splint is indicated he recommends a dorsal splint of some light material. He has used with very satisfactory results whalebone and light strips of steel.

---

DIPHTHERIA.—Dr. James T. R. Davidson, (*British Med. Jour.*, Oct. 25, 1890) reports a summary of investigations, carried out during an epidemic of diphtheria in Buenos Ayres. It is a prevailing custom to publish in the daily papers mortality tables, giving location of residence and cause of death. The doctor concludes from a very thorough investigation that the disease is usually communicated to man by domestic animals, especially hens and horses. He believes animals contract it from living in close, damp quarters, surrounded by decaying animal and vegetable matter.

---

THE UNIVERSITY MEDICAL MAGAZINE.—This very excellent journal has perfected arrangements for making an entirely new departure. The new department will contain from sixteen to twenty additional pages devoted to "medical progress" edited by the following eminent teachers:—Medicine, conducted by William Pepper, M. D., and James Tyson, M. D. Surgery, by D. Hayes Agnew, M. D., and J. William White, M. D. Therapeutics, by Horatio C. Wood, M. D. Gynæcology, by William Goodell, M. D. Obstetrics, by Barton Cooke Hirst, M. D.

Exchanges of the *University Medical Magazine* will, after due consideration, be "deposited upon the shelves of the library of the University of Pennsylvania, which is accessible at all times to the public."

The *Magazine* is one of the best monthly journals published in this country, and this new departure cannot fail to be appreciated.

